# Soil CO_2_ Dynamics in a Tree Island Soil of the Pantanal: The Role of Soil Water Potential

**DOI:** 10.1371/journal.pone.0064874

**Published:** 2013-06-10

**Authors:** Mark S. Johnson, Eduardo Guimarães Couto, Osvaldo B. Pinto Jr, Juliana Milesi, Ricardo S. Santos Amorim, Indira A. M. Messias, Marcelo Sacardi Biudes

**Affiliations:** 1 Institute for Resources, Environment and Sustainability, University of British Columbia, Vancouver, British Columbia, Canada; 2 Department of Earth, Ocean and Atmospheric Sciences, University of British Columbia, Vancouver, British Columbia, Canada; 3 Department of Soil Science, Federal University of Mato Grosso, Cuiabá, Mato Grosso, Brazil; 4 Programs in Tropical Agriculture and in Ecology and Biodiversity, Federal University of Mato Grosso, Cuiabá, Mato Grosso, Brazil; 5 Department of Environmental Biophysics, Federal University of Mato Grosso, Cuiabá, Mato Grosso, Brazil; DOE Pacific Northwest National Laboratory, United States of America

## Abstract

The Pantanal is a biodiversity hotspot comprised of a mosaic of landforms that differ in vegetative assemblages and flooding dynamics. Tree islands provide refuge for terrestrial fauna during the flooding period and are particularly important to the regional ecosystem structure. Little soil CO_2_ research has been conducted in this region. We evaluated soil CO_2_ dynamics in relation to primary controlling environmental parameters (soil temperature and soil water). Soil respiration was computed using the gradient method using *in situ* infrared gas analyzers to directly measure CO_2_ concentration within the soil profile. Due to the cost of the sensors and associated equipment, this study was unreplicated. Rather, we focus on the temporal relationships between soil CO_2_ efflux and related environmental parameters. Soil CO_2_ efflux during the study averaged 3.53 µmol CO_2_ m^−2^ s^−1^, and was equivalent to an annual soil respiration of 1220 g C m^−2^ y^−1^. This efflux value, integrated over a year, is comparable to soil C stocks for 0–20 cm. Soil water potential was the measured parameter most strongly associated with soil CO_2_ concentrations, with high CO_2_ values observed only once soil water potential at the 10 cm depth approached zero. This relationship was exhibited across a spectrum of timescales and was found to be significant at a daily timescale across all seasons using conditional nonparametric spectral Granger causality analysis. Hydrology plays a significant role in controlling CO_2_ efflux from the tree island soil, with soil CO_2_ dynamics differing by wetting mechanism. During the wet-up period, direct precipitation infiltrates soil from above and results in pulses of CO_2_ efflux from soil. The annual flood arrives later, and saturates soil from below. While CO_2_ concentrations in soil grew very high under both wetting mechanisms, the change in soil CO_2_ efflux was only significant when soils were wet from above.

## Introduction

The Pantanal tropical wetland ecosystem is a low-relief landscape situated in the broad depression of central South America. Covering 160,000 km^2^, the Pantanal is among the world's largest wetlands, and is a major priority for conservation [1]. It is comprised of seasonally-flooded savannas and grasslands, permanently saturated depressions and forested terra-firme topographic rises. Across these diverse landforms, soils are strongly influenced by hydromorphism, with soil profiles most fully developed on the terra-firme rises. Locally known as *cordilheiras*, these rises occur on broad paleolevees (e.g. residual depositional riverbanks remaining on the landscape following migration of the river channel), which are important refuges for terrestrial animals during flood periods [2]. Globally, forested topographic rises within wetlands are referred to as “tree islands”, and are recognized as hotspots for both biogeochemistry and biodiversity [3].

Tree islands occur as patches within wetland complexes and have distinctive hydrologic, edaphic and biological functioning relative to their surroundings [4], which increases the ecological complexity in the landscape [5]. Tree islands are a known biogeochemical hotspot in the Florida Everglades [6]. Changes to regional groundwater flow patterns due to construction of canals and levees in the Everglades have resulted in the complete loss of more than 50% of tree islands in recent decades with remaining tree islands suffering degradation with ecological complexity declining as a result [6]. Tree islands in other Neotropical wetlands including the Pantanal are generally less well studied than those of the Everglades.

Biological processes in the Pantanal are strongly moderated by hyperseasonal environmental conditions, e.g. conditions within an annual cycle that are characterized by two contrasting stressors [7]. Within the Pantanal, this hyperseasonality is experienced as a pronounced flooding period with more than 2 m of standing water in many areas [8], [9], followed by an extensive dry-season typically lasting six-months. During the resulting low-water period, spatially-extensive land-use activities are common throughout the Pantanal ecoregion, including cattle grazing and agriculture. Tree island soils are a distinct niche within tropical wetland complexes in that they only briefly experience standing water conditions, if at all. Subsoil saturation of the tree island soil can result from both infiltration of precipitation as well as in response to regional water table dynamics due to floodwaters arriving in the vicinity of tree islands from contributing areas within the wetland's regional watershed.

Maia *et al.* [10] found that during the 1970–2002 period, the Pantanal suffered the greatest loss of soil organic carbon (SOC) of any ecotype within the Brazilian “Legal Amazon” (e.g. the entire area of all Brazilian states that contain a portion of the Amazon basin, and thus includes parts of ecoregions that are outside of the Amazon basin including the portion of the Pantanal located in the Brazilian state of Mato Grosso which also includes a portion of Amazon forest). This loss was the greatest of any ecotype considered in the Maia *et al.* [10] study, both on a per hectare basis as well as when considered as a rate of change (e.g. 1985–2002 relative to 1970–1985). Comparing the 1985–2002 period to the 1970–1985 period, annual SOC loss in the Pantanal increased from 0.94 Mg C ha^−1^ yr^−1^ to 1.16 Mg C ha^−1^ yr^−1^. This change in Pantanal SOC stocks is attributed primarily to degradation of the grassland ecosystem related to extensive cattle ranching activities [10], noting that artificial drainage is not practiced in the Pantanal. These dynamics point towards the need for a better understanding of biogeochemical processes in this highly biodiverse environment.

The primary loss pathway for SOC is via decomposition, which results in soil respiration losses of CO_2_ to the atmosphere, as well as translocation of terrestrial respiration products to the hydrosphere with percolating soil water [11] and transport of SOC via erosion and deposition [12]. At the global scale, CO_2_ emissions from wetlands are lower than emissions from non-wetland soils due to the impact of soil saturation, and global wetland emissions tend to correlate with temperature and not with precipitation [13].

However, little is known about soil respiration within tropical wetland complexes [14], [15]. Since temperature regimes in tropical wetlands do not vary substantially over the course of a year, we hypothesized that soil moisture dynamics would play a stronger role than temperature in controlling soil respiration. Since water is involved in both the production of CO_2_ within soil, as well as limiting CO_2_ and O_2_ diffusion within soil and between soil and the atmosphere [16], a dynamic consideration of the relationships involved is needed. Several studies have investigated the dual role that the infiltration front exerts on soil respiration dynamics following rain events plays by increasing CO_2_ concentrations while decreasing diffusivity [17], [18]. In particular, the gradient method for soil respiration provides the opportunity to explore relationships between soil water dynamics and subsurface soil CO_2_ dynamics in conjunction with other soil parameters [19].

In this study, we sought to quantify soil respiration for a terra-firme rise within the Pantanal wetland complex, and to identify the key controls on soil CO_2_ efflux and soil CO_2_ dynamics within the soil profile. Focusing the study on the topographic rise of the tree island enabled consideration of the full range of soil moisture conditions and flooding dynamics (e.g. as regionally controlled via water table dynamics vs. locally controlled by direct precipitation). In particular, the study was designed to evaluate the roles of and interactions between soil moisture content and soil water potential (e.g. soil tension) on soil CO_2_ processes. Relatively few studies to date have explicitly considered the influence that changes in soil water potential can have on soil respiration [20].

## Materials and Methods

### Site Description

**Figure 1:**
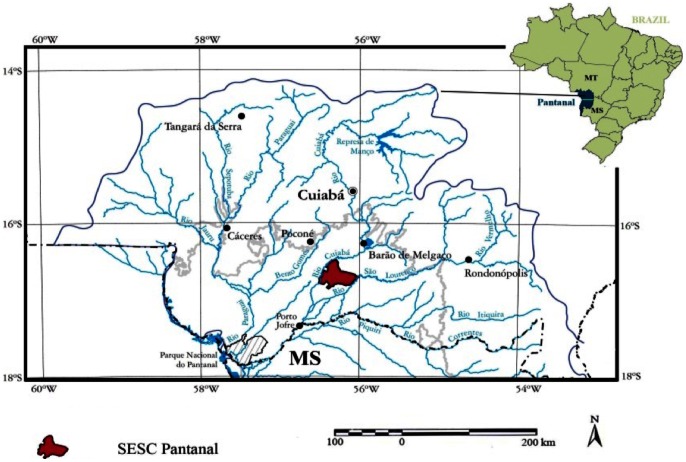
Site location map showing the SESC Pantanal research site within the Northern Pantanal. The inset map shows the location of the Brazilian portion of the Pantanal.

Research was conducted in the northern portion of the Pantanal wetland (56.28°W, 16.57°S) within the long-term ecological research (LTER) station known as SESC-Pantanal near Poconé, Mato Grosso, Brazil (Figure 1). SESC-Pantanal is included in the Ramsar Convention list of Wetlands of International Importance, and is managed by the Brazilian Social Service of Commerce (SESC) [21]. Soils in the region show a high degree of hydromorphism [22]. The soil profile studied in this investigation was classified as a Haplic Planosol in the FAO Classification, with sand and clay contents of 90% and 7% respectively throughout the upper 85 cm, with the clay content increasing to 40% only below 1 m depth. Soils are acidic (pH 4.6 in 1∶2.5 slurry of soil:water), with very low (<1%) organic carbon (C) contents. Precipitation averages about 1250 mm y^−1^, with an extensive dry season lasting from May through September. Due to the hyperseasonality of the study area, the soil moisture regime for the tree island soil is classified in the U.S. soil taxonomy [23] as Ustic in the dry season and Aquic in the wet season.

The overstory canopy is dominated by *Curatella americana* L. (known locally as *lixeira*) and *Dipteryx alata* (known locally as *cumbaru*), with an understory dominated by *Scheelea phalerata* (Mart.) [9], which is locally referred to as *acuri* palm. The site has a leaf area index of 3.5 m^2^ m^−2^, a stand density of 1130 trees ha^−1^, and a basal area of 0.2 m^2^ h^−1^ [9]. Vourlitis et al. [9] measured the soil surface litter pool at 1.25 kg m^−2^, which is equivalent to 600 g C m^−2^ assuming a carbon density of 0.48 g C per g dry litterfall [24]. Litterfall is highly seasonal for this semi-deciduous forest type, peaking during the dry season [25].

### Field measurements

Field measurements were carried out from late November 2008 through March 2010. Environmental sensors were installed within a soil profile in clusters at 10 and 30 cm depths within the soil profile. The location of the soil profile was chosen to characterize the tree island by selecting a central location on the island that was neither a topographic high nor low. The 10 cm and 30 cm sensor clusters were staggered laterally (e.g. offset horizontally) by 1.5 m such that the installation would not impede nor enhance the movement of water, heat or soil gases between measurement depths. Each cluster consisted of sensors to measure temperature, soil water content, soil water potential (e.g. soil tension), and the carbon dioxide concentration of the soil air. Within each cluster, sensors were separated laterally by 10 cm to avoid interferences. Volumetric soil water content was measured using probes that utilize a capacitance/frequency domain approach (model EC-5, Decagon Instruments, Pullman, Washington, USA). Soil water potential was analyzed using dielectric water potential sensors (model MPS-1, Decagon Instruments, Pullman, Washington, USA). Soil CO_2_ concentrations were determined *in situ* using infrared gas analyzers (model GMM221, Vaisala Inc., Helsinki, Finland) [26], [27].

Soil water parameters (soil water content (Θ in cm^3^ cm^−3^) and soil matrix potential (e.g. soil water potential, Ψ in kPa)), barometric pressure, logger panel temperature and battery voltage were measured at 30 second intervals with averages recorded every thirty minutes. The data logger (Campbell Scientific model CR-1000, Campbell Scientific International, Logan Utah) controlled the soil water sensor excitation and the on-off cycling of the CO_2_ sensors. The CO_2_ sensors were powered up for five minutes during each half-hour due to power consumption of 4W per sensor [28]. The first three minutes of each power-on period corresponded to the sensor warm-up period, with readings made each 30 seconds during the subsequent two minutes of the power-on period. The resulting soil CO_2_ readings were then averaged and recorded on a half-hourly basis.

Barometric pressure and soil temperature measurements (BPS sensor and soil thermister, Apogee Instruments, Logan, Utah, USA) were used to correct soil CO_2_ readings due to pressure and temperature dependencies of sensor output related to the ideal gas law [28]. Previously published studies [27] presented a temperature correction equation for use with Vaisala CO_2_ sensors that results in an erroneous temperature correction factor when CO_2_ concentrations are very high, as is the case in the present study. This is due to the inclusion of raw CO_2_ concentration as a variable in the third-order polynomial temperature correction equation of Tang *et al.* [27]. Rather, we employed temperature and pressure correction terms that were presented in the more recent equipment manual [28].

The data logger was connected to a large (85 Ah) capacity 12V DC battery and a 20W solar panel and charge regulator. The solar panel was deployed in a clear-sky area, allowing the system power to be maintained by the solar panel. The large capacity of the battery ensured continuous power to the system during nights and extended cloudy periods of reduced solar radiation. At no point during the study did the battery voltage drop below 12V.

The soil CO_2_ sensors were calibrated with a measurement range of 0–100,000 ppm CO_2_ (0–10% CO_2_), with a maximum reading of 115,000 ppm CO_2_. This range was selected based on previous experience in the Brazilian Amazon [11]. Measurements exceeding the sensor range were gap-filled using the maximum sensor reading of 115,000 ppm in order to produce a continuous record for time-series analysis. About 5.1% of CO_2_ observations at 30 cm were gap-filled, which increased the annual flux estimate by 8% compared to the non-gap filled data. At the 10 cm depth, the CO_2_ readings were always within the measurement range of the sensor.

### Calculating soil respiration CO_2_ flux

CO_2_ efflux from the soil surface (e.g. soil respiration) was calculated at each 30 minute time step using the flux gradient method [17], [27]. This approach is based on Fick's fist law of diffusion, where the CO_2_ fluxes within the soil profile are calculated at two or more depths as:
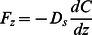
(1)where the *F_z_* is the flux (µmol m^−2^ s^−1^) at depth z (m) determined based on *dC*, the change in CO_2_ mole concentration (µmol m^−3^) over the depth interval *dz* (m), and the diffusivity of CO_2_ in soil, *D_s_* (m^2^ s^−1^), at depth z. As the Vaisala sensors produce concentrations as volume fractions (µmol mol^−3^), the output must be transformed to mole concentrations based on the ideal gas law as described in Tang *et al.* [27].

Soil respiration (*F*_0_) is computed after Vargas *et al.* (2010) from the fluxes within the soil profile as:
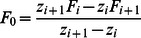
(2)where the soil CO_2_ fluxes at depths z = i and i+1 are determined from equation (1) and the soil respiration efflux (*F*_0_) corresponds to z = 0. In the present study, CO_2_ fluxes within the soil profile were calculated for z_i+1_ = 20 cm and z_i_ = 5 cm and using CO_2_ concentrations at 10 cm and 30 cm for *F*_20_, and soil CO_2_ at 10 cm and atmospheric CO_2_ for *F*_5_. Atmospheric CO_2_ concentration values (*C_air_*) were obtained at 2 m above the soil surface using a Li-cor LI-6400XT infrared gas analyzer mounted below the forest canopy on a flux tower located within 1 km of the tree island where the soil CO_2_ sensors were installed (M. Biudes, unpublished data).

Soil diffusivity, *D_s_*, was computed at each timestep based on an empirically derived relationship between volumetric soil water content (Θ) and CO_2_ diffusivity in soil determined in the laboratory. For diffusivity measurements, large rings (10 cm diameter and 10 cm height) were used to collect undisturbed soil samples from 5–15 cm (n = 3) and 25–35 cm depths (n = 2) corresponding to IRGA installation depths. The samples were brought from the field to the lab at UFMT where soil diffusivity measurements were conducted according to Jassal *et al.* [29] for water contents ranging from 0.04–0.38 cm^3^ cm^−3^. Here, a gas mix of CO_2_ in air (5% CO_2_) was introduced into the base of a chamber that housed an additional Vaisala CO_2_ sensor. The chamber base also provided a support for the soil sample. A barometric pressure sensor was placed in the lower chamber to ensure that pressure in the chamber remained at atmospheric. The chamber was equipped with an unobstructed exit port to allow free flow of calibration gas out of the chamber while a portion of the calibration gas diffused upwards into the soil sample. The calibration gas was vented from the exit port to an outside window to avoid build up of CO_2_ in the laboratory environment.

We used an additional field sample to determine the length of time required to reach steady-state conditions (e.g. constant *F*_0_) by making repeated measurements of *F*_0_ at the sample surface using a Li-cor soil CO_2_ flux chamber coupled to a Li-cor LI-6400XT gas analyzer. Steady-state conditions were achieved after allowing the calibration gas to diffuse for an hour. From there, D_s_ was calculated after Jassal *et al.* (2005) as:
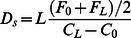
(3)where L is the length of the soil sample, *F*_0_ is soil respiration at steady-state, *F_L_* is the CO_2_ flux at the bottom of the soil sample, and C_0_ and C_L_ are the CO_2_ concentrations at the top and bottom of the soil sample at steady state, respectively. C_0_ was measured with the Li-cor LI-6400XT, and C_L_ was measured with the Vaisala sensor. *F*_L_ resolves algebraically to *F*_0_ – *F*_s_, where *F*_s_ is the flux of CO_2_ generated within the soil sample (e.g. the background soil CO_2_ production rate). *F*_s_ was measured on each soil sample prior to each diffusivity measurement by capping the bottom of the sample and measuring soil efflux with the Li-cor analyzer prior to placing the sample on the diffusivity measurement chamber. *F*_s_ is then substituted into equation (3) as *F*_L_  =  *F*_0_ – *F*_s_ [29].

Soil samples were weighed prior to each diffusivity measurement, and soil water content was determined for each measurement based on the difference between measurement weight (mass of wet soil minus ring weight) and the oven-dry soil mass. The latter was determined after concluding diffusivity measurements. For soil collected from 5–15 cm depth (e.g. centered on the CO_2_ sensor installed at 10 cm depth), the relationship between soil volumetric water content (VWC, cm^3^ cm^−3^) and diffusivity of CO_2_ in soil (m^2^ s^−1^) was found to be 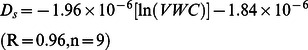
(4)


For the 30 cm depth (e.g. soil samples collected from 25–35 cm), the following relationship was found: 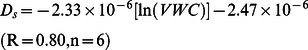
(5)


Soil efflux computed from the gradient method [17] was compared against field-based soil efflux measurements using Li-cor soil CO_2_ flux chamber kit (model 6400–19) coupled to a Li-cor LI-6400XT gas analyzer on six different measurement days. There was a significant relationship between the gradient-calculated soil CO_2_ efflux and that measured in the field:
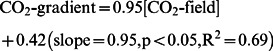
(6)


### Statistical analyses

Initial data exploration was conducted via factor analysis in order to identify interdependencies between measured parameters (e.g. common factors), and to identify temporal clusters within the reduced parameter space. Factor analysis techniques have frequently been used successfully with soil data [30]. We applied factor analysis using a varimax rotation that included all measured variables (soil CO_2_ concentration, soil temperature, soil moisture content and soil water potential), treating parameters at each depth as independent variables. The varimax method rotates the significant axes of resulting factors orthogonally in order to force the loadings of the original components of each factor to be either as large as possible, or near zero [31]. This procedure has the advantage of simplifying the interpretation of the resulting factors, and has been utilized in research related to soil, water and meteorology [32], [33]. The factor analysis was run in SPSS v17 (IBM Corp., New York).

Linear correlations were determined between all measured parameters. For water potential, correlations were determined for both the sensor output in kPa as well as in units of pF calculated as:
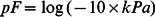
(7)where kPa is expressed as tension (e.g. negative values). Soil water potential is often expressed in its logarithmic form (pF) due to the nonlinear relationship between soil water potential and soil moisture content, as well as due to the log-linear relationship between soil water potential and soil respiration [20].

The relationships between soil CO_2_ efflux and its principal controlling variables (soil water and soil temperature) were evaluated for the full study period using wavelet coherency analysis [34]. Here, we determined the coherence of the variance between two variables in the frequency domain, analyzing independently for soil CO_2_ efflux vs. soil water potential, and again for soil CO_2_ efflux vs. soil temperature. Soil water potential was utilized in the time series analyses as it was measured independently from soil moisture content, and was not used in the calculation of soil CO_2_ efflux. Wavelet coherency analysis was conducted in the R software environment for statistical computing and graphics (R Version 2.15.3) [35] using the R package biwavelet (Version 0.13) [36]. For wavelet coherency analysis, we used a total time series length of 270 days, which encompassed 90 days for each of the temporal clusters identified by factor analysis.

We then analyzed each of the temporal clusters independently using conditional nonparametric spectral Granger causality analysis [37], [38]. In this approach, causal relationships between the dependent variable (here, soil CO_2_ efflux) and independent variables are evaluated in the frequency domain for a multivariate system based on established principles known as Granger causality, or G-causality [39], [40], [41]. We analyzed soil CO_2_ efflux in relation to soil water potential and soil temperature for a 90-day period for each temporal cluster after porting data from R to Matlab using the R package R.matlab [42]. We used the Granger causality Matlab toolbox available for download [37], which we ran in Matlab R2011b.

Briefly, Granger causality is based upon the logical assumption that causes must precede effects. For Granger causality analysis, a series of t tests and F tests are employed on lagged time series data to quantify if there is information in the presumed causal variables that contribute to the variability of the presumed response variable. For conditional nonparametric spectral Granger causality analysis, an individual variable can be analyzed for causality while controlling for a second potential causal variable. For example, in a system with two potentially controlling variables (X_1_ and X_2_), the influence of X_1_ and X_2_ on the outcome variable (Y) can be evaluated individually; one of the independent variables (X_1_) is first evaluated for G-causality in the frequency domain, and is then is reevaluated while controlling for the other independent variable (X_2_). If the G-causality between X_1_ and Y is significant while controlling for X_2_, the relative strength of the control of X_1_ on Y can be assessed across a frequency spectrum. This is then repeated to assess the strength of the influence of X_2_ on Y (with and without controlling for X_1_). Complete details on the method have been presented previously in the literature [37], [38].

### Permissions

No specific permits were required for the described field studies, which took place within the SESC Pantanal Reserve. Research within the reserve is coordinated as part of the Brazilian Long-term Ecological Research (LTER) network by the Federal University of Mato Grosso. The field studies did not involve any endangered or protected species.

## Results

### Factor analysis and identification of seasons

**Figure 2:**
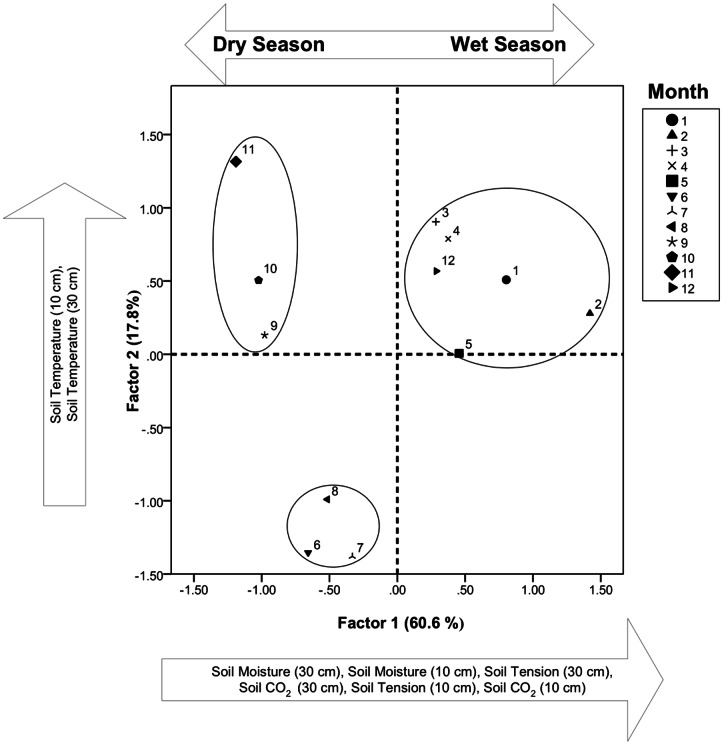
Factor analysis results. The circles enclose months with similar variance in the measured parameters. These are referred to in the paper as seasonal clusters.

Factor analysis resulted in two principal factors. Soil water parameters (soil water potential and soil moisture content) and soil CO_2_ from both 10 cm and 30 cm depths comprise Factor 1, which explained slightly more than 60% of the total variance in the parameter set (Figure 2). Factor 2 was limited to soil temperature, which explained an additional 17.8% of the total variance. Results from the factor analysis were aggregated and plotted within the two-factor space by month, which resulted in three distinct clusters of data (Figure 2). The largest cluster was found to consist of wet season months, with the other two clusters split between cooler and warmer months during the dry season. These clusters correspond to the hydrologic periods of the northern Pantanal, which is at maximum flood during February, with lowest water in August [2]. The clusters, while primarily descriptive, were useful for distinguishing seasonal behavior within the system. We also used them to identify episodes of similar magnitude occurring in distinct seasons, which we present in a subsequent section of the paper.

A correlation matrix of variables utilized in the factor analysis is presented in Table 1. For CO_2_ concentration in the near-surface layer (10 cm), the strongest correlation obtained among other near-surface (10 cm) parameters was for soil water potential, followed by soil moisture (Table 1). At 30 cm depth, CO_2_ concentration was most highly correlated with 30 cm measurements of soil moisture followed by soil water potential. Soil temperature was weakly correlated with soil CO_2_ concentrations at both depths. The depth-dependent differences in the relationships between soil water potential, soil moisture and soil CO_2_ concentrations are explored further in a subsequent section.

**Table 1:** Correlation matrix of measured parameters.

	Soil CO_2_ efflux	Soil CO_2_ (10 cm)	Soil CO_2_ (30 cm)	Soil temp. (10 cm)	Soil temp. (30 cm)	Soil water (cm^3^ cm^−3^, 10 cm)	Soil water (cm^3^ cm^−3^, 30 cm)	Soil tension (kPa, 10 cm)	Soil tension (kPa, 30 cm)	Soil tension (pF, 10 cm)	Soil tension (pF, 30 cm)
Soil CO_2_ efflux	1	0.93	0.36	0.14	0.26	0.32	0.45	0.52	0.41	−0.57	−0.45
Soil CO_2_ (10 cm)		1	0.53	0.12	0.22	0.49	0.57	0.52	0.48	−0.61	−0.53
Soil CO_2_ (30 cm)			1	0.27	0.35	0.81	0.81	0.61	0.65	−0.71	−0.73
Soil temp. (10 cm)				1	0.89	0.21	0.25	0.21	0.26	−0.2	−0.28
Soil temp. (30 cm)					1	0.31	0.35	0.35	0.35	−0.33	−0.37
Soil water (cm^3^ cm^−3^, 10 cm)						1	0.8	0.69	0.63	−0.79	−0.74
Soil water (cm^3^ cm^−3^, 30 cm)							1	0.74	0.94	−0.8	−0.98
Soil tension (kPa, 10 cm)								1	0.69	−0.95	−0.76
Soil tension (kPa, 30 cm)									1	−0.71	−0.97
Soil tension (pF, 10 cm)										1	0.79
Soil tension (pF, 30 cm)											1

Values correspond to Pearson's correlation coefficient (R). P<0.05 for all correlations in the matrix.

### Soil CO_2_ dynamics

**Figure 3:**
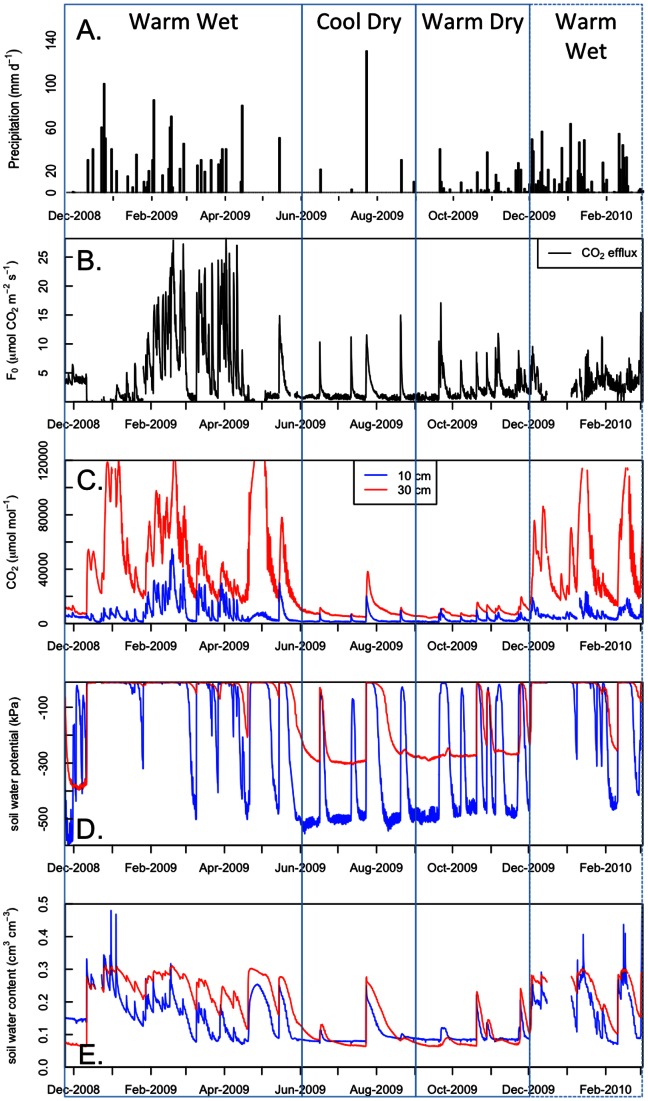
Precipitation (A), soil respiration (B), soil CO_2_ concentrations at 10 cm and 30 cm depths (C), soil water potential (D) and soil water content (E) during the December 2008 -December 2009 study period. Vertical boxes indicate the seasonal periods identified by factor analysis.

Mean soil CO_2_ concentrations during the period of study were 4940 ppm at 10 cm and 27630 ppm at 30 cm. Soil CO_2_ concentrations and soil respiration were strongly seasonal (Figure 3), with highest values during the wet season and lowest during the dry season. This broad seasonal trend is overlain by soil CO_2_ responses to wetting episodes in both wet and dry seasons, with soil CO_2_ at 10 cm (Figure 3c) exhibiting a more dynamic response than soil CO_2_ at 30 cm (Figure 3c). Soil efflux during the study averaged 3.53 µmol CO_2_ m^−2^ s^−1^. Annual soil respiration was 1220 g C m^−2^ y^−1^. As the study period encompassed November 2008 through March 2010, we averaged the values for the days of year that occurred more than once in the time series to avoid seasonal biases (e.g. values for February 2, 2009 and February 2, 2010 were averaged before computing annual means).

In order to evaluate the sensitivity of calculating soil CO_2_ efflux via the gradient approach using CO_2_ values for *C_air_* that were measured 2 m above the soil surface within 1 km of the location of the soil measurements, we recalculated soil CO_2_ efflux using the average global CO_2_ concentration during the period of study for *C_air_* (386 ppm) [43]. The resulting value was within 0.7% of the value determined when using the tower-measured CO_2_ concentrations. Computing soil CO_2_ efflux with the gradient approach using a constant value for *C_air_* introduces minor systematic errors at diurnal and seasonal time scales, although these errors tend to cancel over the course of a year [44]. In general, since soil CO_2_ at 10 cm was typically one to two orders of magnitude greater than *C_air_*, variations in *C_air_* have little impact on the steep gradient between soil CO_2_ and *C_air_* [44].

### Soil water dynamics

Soil moisture and soil water potential also exhibited broad seasonal trends, which were interspersed with episodic wetting events during the dry season, and episodic drying events during the wet season (Figure 3d and e). During the wet season, soils were generally at or near saturation with periodic drying events observed during which soil CO_2_ concentrations declined rapidly. During the dry season, soil water was typically at minimal levels for soil water potential and soil moisture content measurements, with periodic wetting events observed that corresponded to rapid increases in soil CO_2_ concentrations. Generally, soil CO_2_ concentrations were highest following extended periods with soil water potential values near zero (Figure 3).

**Figure 4:**
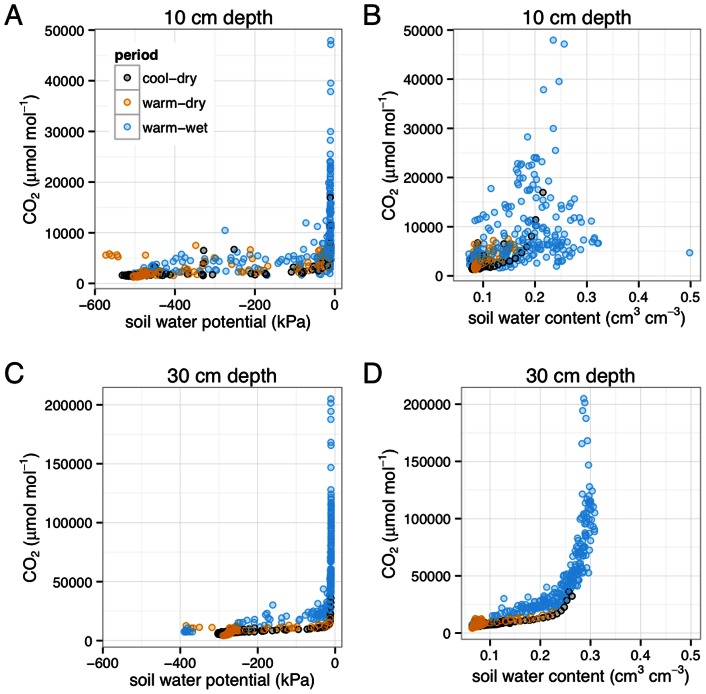
Soil water parameters vs. CO_2_ concentrations at 10 cm and 30 cm depths, plotted as daily averages to reduce overplotting.

The relationships between soil water parameters and soil CO_2_ concentrations differed for soil moisture as compared to soil water potential. Soil water potential had a bimodal behavior in this coarse-textured soil, dropping rapidly to very negative values when soils drained, and climbing rapidly to near-zero values following precipitation and infiltration events (Figure 4a and 4c). High CO_2_ values were observed only under moist conditions when soil water potential approached zero. Below a threshold soil water potential of approximately −0.15 kPa, changes in soil CO_2_ were fairly limited, suggesting that physiological moisture stress of roots or soil microbes were not the dominant drivers of soil CO_2_ throughout the year. At the 30 cm depth soil CO_2_ increased exponentially with moisture to the saturation point (0.3 cm^3^ cm^−3^). The relationship between CO_2_ concentration and soil diffusivity at 30 cm was strongly linear (r^2^ = 0.53, p<0.001), suggesting that diffusivity restricts CO_2_ transport at high soil moisture contents, which leads to transient storage of CO_2_ in soil. At the more porous 10 cm depth, soil CO_2_ increased with moisture to an intermediate water content (∼0.25 cm^3^ cm^−3^), but decreased at higher water contents (Figure 4b). The declining values in soil CO_2_ at higher levels of soil moisture suggests diffusive limitations on gas transport that could reduce CO_2_ efflux or O_2_ influx. We also observed respiration pulses at both 10 cm and 30 cm depths for soils that were initially quite dry (c.f. soil water dynamics in Figure 4a and 4c relative to CO_2_ efflux in Figure 3b). This is an example of discrete events leading to dynamics that deviate from the gross seasonal grouping identified in the factor analysis.

### Time series analysis of soil CO_2_ efflux relative to soil water potential and soil temperature

**Figure 5:**
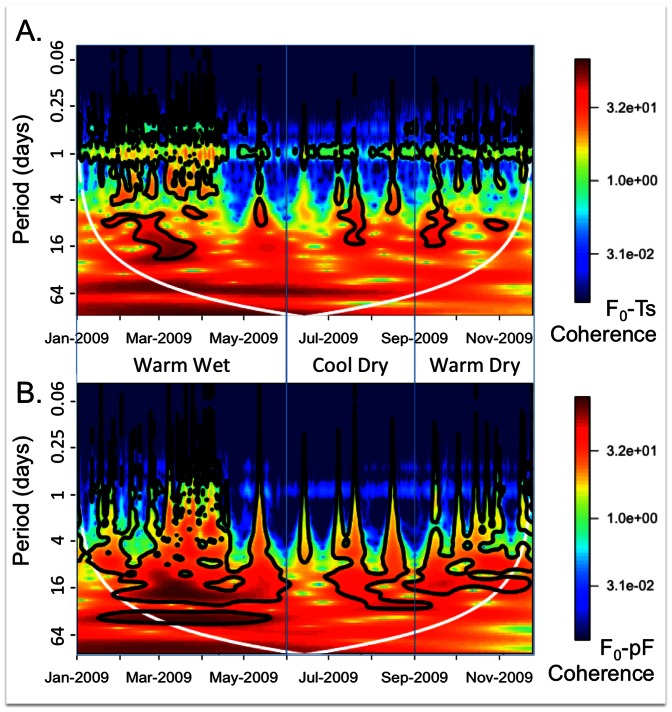
Wavelet coherence between soil CO_2_ efflux (F_0_) and soil temperature (T_s_) (A, upper panel), and between F_0_ and soil water potential (pF) (B, lower panel). The thick black lines outline the periods that are statistically significant at the 0.05 level. The variance in the soil CO_2_ efflux and soil temperature time series exhibited statistically significant coherence at the 24-hour timescale, with coherence between soil CO_2_ efflux and soil water potential strongest for bi-weekly timescales.

The wavelet coherence spectra between soil CO_2_ efflux and soil temperature exhibited significant coherency at the daily timescale across most of the study period (Figure 5a). The coherence between soil CO_2_ efflux and soil water potential showed greater power in response to wetting episodes, which persisted over multi-day to bi-weekly timescales (Figure 5b). This suggests that soil water potential could be a more important control on soil CO_2_ efflux than soil temperature for this system for longer timescales, although there is also coherence between soil CO_2_ efflux and soil water potential at the daily timescale that was not statistically significant. However, in wavelet coherency analysis, it is not possible to control for potential correlations between causal factors (such as soil temperature and soil water potential).

**Figure 6:**
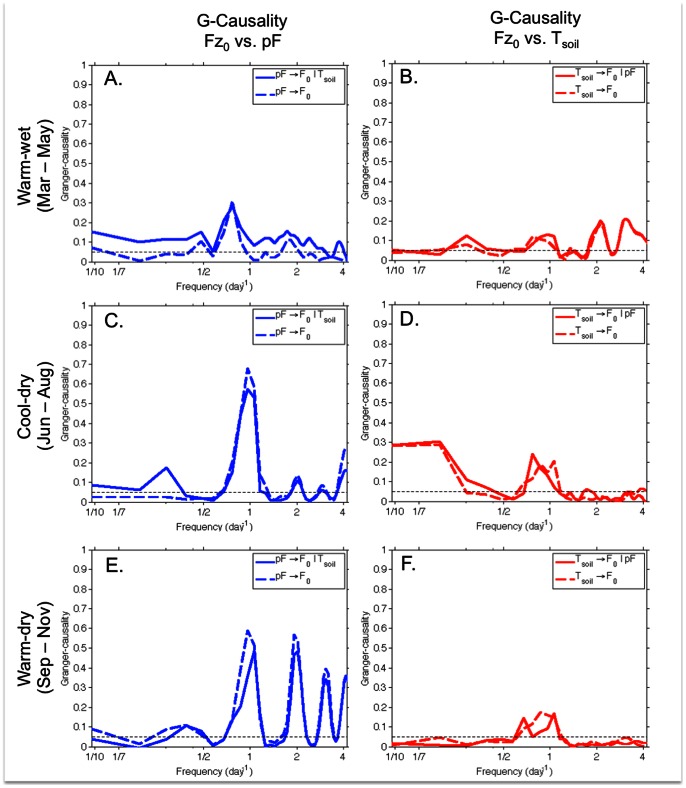
Granger-causality spectra between soil water potential (pF) and soil temperature (T_s_) on soil CO_2_ efflux (F_0_). Results are presented for each temporal cluster identified in the factor analysis, with 90-day time series used for each cluster.

We then employed conditional nonparametric spectral Granger causality analysis to explore the strength of soil water potential (pF) and soil temperature (T_s_) as controls on soil CO_2_ efflux (F_0_) when controlling for correlations between the independent variables pF and T_s_. During the warm-wet period, T_s_ was found to significantly G-cause F_0_ at the daily timescale and when conditioned on pF (Figure 6b). When pF was conditioned on T_s_, it was also found to significantly G-cause F_0_ at the daily timescale during the warm-wet period (Figure 6a), although neither factor was particularly strong as a causal factor at the daily timescale for the warm-wet period. For this period, T_s_ showed stronger G-causal power for F_0_ at higher frequencies (e.g. at 12-hour and 8-hour timescales, equivalent to 2 times per day and 3 d^−1^), with pF exhibiting stronger G-causality at lower frequencies (e.g. longer timescales; Figure 6b).

The strongest evidence for pF as a more significant casual factor for F_0_ compared to T_s_ was observed during the cool-dry period, when G-causality was highest for pF G-causing F_0_ conditioned on Ts at the daily timescale (Figure 6c). Ts was a significant G-causal factor of F_0_ at lower frequencies during the cool-dry period (Figure 6d). pF conditioned on T_s_ was also found to be the strongest control on F_0_ during the warm-dry period at daily timescales and higher frequencies (e.g. shorter timescales; Figure 6e). T_s_ conditioned on pF was a weaker, though still significant G-cause of F_0_ at the daily timescale for the warm-dry period (Figure 6f).

### Coupled seasonal dynamics between soil respiration and soil hydrology

The strongly seasonal nature of climatic conditions in the Pantanal results in a significant coupling between carbon and water cycling. At the start of the study period, the soil was dry with low soil CO_2_ concentrations and minimal soil respiration. During the wet-up period, the soil quickly reached saturated and near saturated conditions (Figure 3). This resulted in the accumulation of a substantial amount of CO_2_, particularly at the 30 cm depth where values in excess of 80,000 ppm were recorded for several weeks. Despite the high concentration at depth, the efflux of CO_2_ from the soil remained low due the high water content and hence low diffusivity preventing movement of CO_2_ from lower in the profile to the soil surface.

During the wet-up, there were a few excursions from saturation in the surface soil layer. For these periods when the surface soil was less than saturated, precipitation resulted in CO_2_ efflux events due to CO_2_ production in the surface soil. Only once the soil CO_2_ concentration in the surface soil was sustained at a high level did the soil CO_2_ efflux reach maximum (e.g. January 2009 in Figure 3b). Drying conditions observed in the soil water content data (e.g. February 2009 in Figure 3e) resulted in declines in soil CO_2_ concentrations and soil CO_2_ efflux. However, only once soil water potential showed clear evidence of drying did soil CO_2_ efflux exhibit drastic declines. This drying event during the wet season is explored further in the next section.

### Episodic behavior of soil respiration

**Figure 7:**
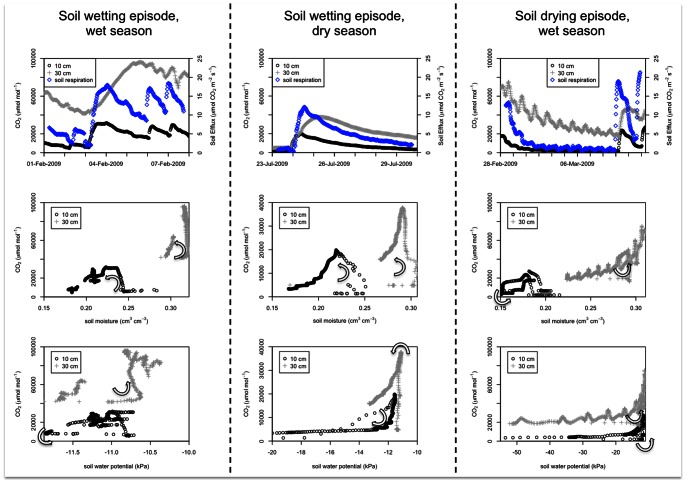
Episodic behavior of soil respiration in response to soil water potential and soil moisture content at 10 and 30 cm depths. The arrows in the lower panels indicate the temporal direction of hysteresis loops.

Soil CO_2_ concentrations and soil efflux responded rapidly to precipitation events (Figure 7). We selected three time slices to illustrate wetting and drying processes, with one illustration provided for each seasonal cluster described in Figure 2. During the wet season, numerous discrete precipitation events are observed at 10 cm depth (Figure 7 upper left panel). The rapid responses of soil CO_2_ and soil respiration were dampened at 30 cm depth. During the dry season, the more discrete nature of wetting events resulted in very pronounced responses in soil CO_2_ concentrations at 10 cm and 30 cm, although the response at 30 cm lags that of 10 cm by 24 hours (Figure 7, upper panel of the center column). A period without rainfall during the wet season allowed for consideration of soil CO_2_ dynamics during a “drying episode”. Here (Figure 7, upper right panel), soil CO_2_ clearly demonstrates diurnal periodicity which is stronger at 30 cm than at 10 cm. Hysteresis patterns in CO_2_ versus soil water parameters of the lower two rows of panels in Figure 7 are considered later in the paper.

## Discussion

### Carbon Fluxes in a Tropical Hyperseasonal Wetland Soils

Soil respiration was calculated as 1220 g C m^−2^ y^−1^ efflux of CO_2_ from soil during the study period. These are the first known values of soil respiration for any Pantanal soil. Further, there are no data at present for any non-carbon accumulating (e.g. non-peat) tropical wetland soils in the Soil Respiration Database (SRDB) described in Bond-Lamberty & Thomson [15] (version 20120510a was consulted during the writing of this paper).

Soil organic carbon (SOC) stocks in the upper soil are low, and approximately equivalent in magnitude to the carbon efflux from soils. Of the 1400 g C m^−2^ for the 0–20 cm of soil [45], the upper 10 cm of soil contained 940 g C m^−2^, with the 10–20 cm depth containing an additional 460 g C m^−2^, which is consistent with other studies of SOC in the Pantanal [9], [46]. In a separate study, the carbon stock in surface litter was found to be about 600 g C m^−2^ during the dry season [9].

Despite the recurrent saturation at or near the soil surface and occasional shallow inundation, the hyperseasonality of the Pantanal inhibits the accumulation of carbon in soils. This non-accumulating nature of soil carbon in the Pantanal directly differs from the general characteristics of wetland soils with large carbon stocks and low fluxes, including tropical and semi-tropical wetland systems such as the Florida Everglades and tropical peat wetlands in Indonesia [47].

For much of the study period, the soil moisture is seen to respond to local precipitation, which is expressed in the rapid rise and slow recession in the soil water content time series (Figure 3E). The exception occurred during May 2009 when regional flooding led to a rising water table that saturated the soil from below, resulting in high CO_2_ concentrations but low soil CO_2_ effluxes. Regional controls on soil moisture via flooding would then be expected to limit turnover of soil carbon, while local controls on soil moisture via precipitation infiltration leads to rapid turnover of soil carbon.

### Soil water potential as primary control on soil CO_2_ dynamics in the Pantanal tree island soil

Respiration increases following wetting are known as the “Birch effect”, and are well documented in the literature [48], [49]. Kim *et al.* [50] recently surveyed the literature related to soil gas responses to rewetting events, and noted a number of biological and physical mechanisms potentially responsible. Biological priming mechanisms identified include the accumulation of substrate during dry periods for subsequent microbial metabolism, and enhanced root exudation following rewetting that primes microbial metabolism [50]. Physical mechanisms included disruption of soil aggregates, and reduced diffusivity following rewetting [50]. Transient storage of CO_2_ also occurs in soil when diffusivity limits soil CO_2_ efflux [51], [52].

**Figure 8:**
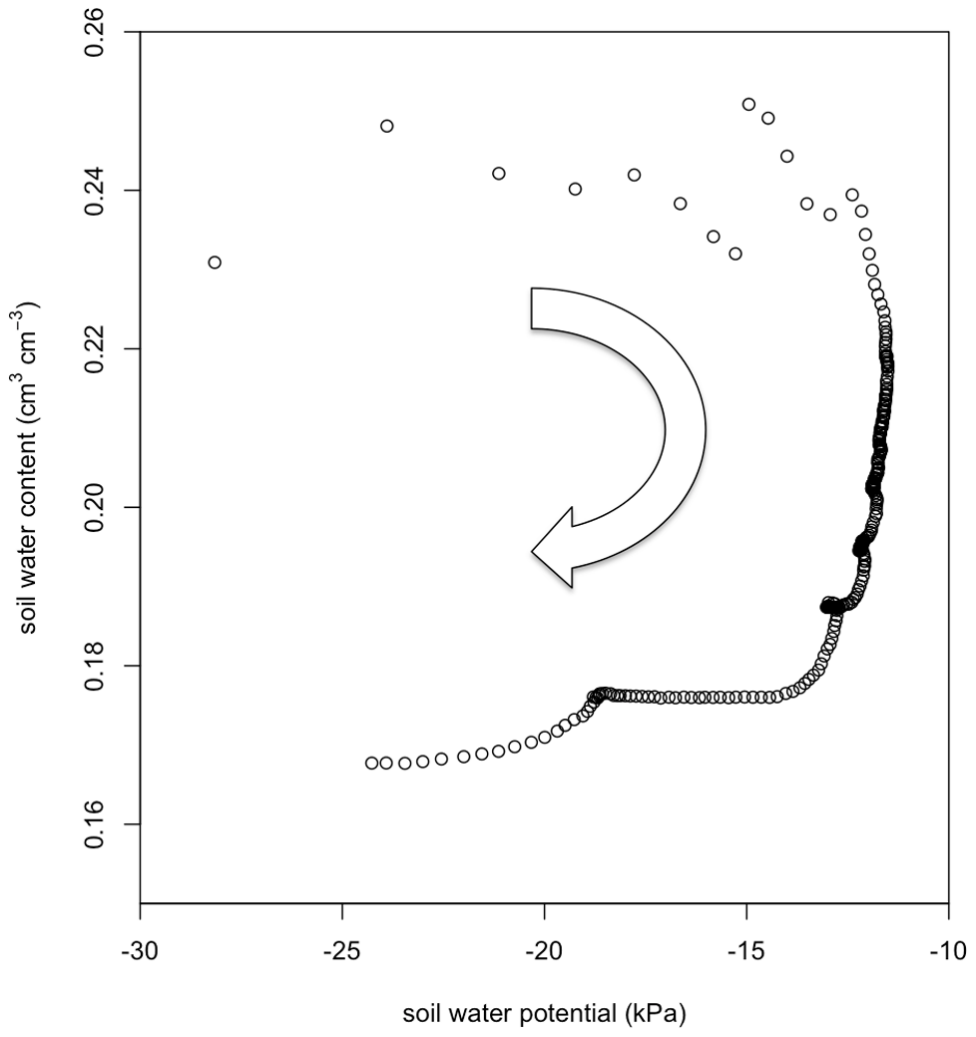
Hysteresis between soil water potential and soil water content at 10 cm depth during a wetting event during the dry season. The arrow indicates the temporal progression during the event.

The overwhelming majority of soil respiration studies evaluating soil water controls on CO_2_ efflux have evaluated soil water content without independently considering soil water potential (with exceptions including Bauer *et al*. [53], Fisher [49] and Orchard and Cook [54]). This is largely due to methodological factors, as soil moisture content has long been a simpler measurement relative to soil water potential [20]. While some studies have computed soil water potential from measurements of soil moisture content in conjunction with the soil water characteristic [55], this approach does not permit consideration of hysteresis in the relationship between soil water potential and soil moisture content. Hysteresis as it refers to soil water behavior is the non-monotonic behavior of the relationship between soil moisture content and soil water potential [56]. That is, the relationship between soil moisture content and soil water potential differs significantly between the wetting and drying phases, which also affects soil respiration. For example, clockwise hysteresis between soil moisture content and soil water potential (Figure 8) was observed during the wetting event that occurred during the dry season depicted in Figure 7 (center column). Soil water content varied by 50% during the event at −20 kPa soil water potential (Figure 8), indicating the importance of independent measurements of the two soil water properties.

Previous studies in temperate zone soils have found hysteresis between soil respiration and soil temperature [57], [58]. However, due to the minor variance in soil temperature in the Pantanal study area, soil temperature did not demonstrate any consistent hysteresis patterns with soil respiration. Rather, hysteresis was observed between soil respiration and soil water measurements (Figure 7). For wetting events, soil CO_2_ increased rapidly once soil water reached a critical level, and then declined slowly as soil dried (e.g. counter-clockwise hysteresis). The one exception was for soil CO_2_ vs. soil water potential at 10 cm during the dry-season wetting episode. There, soil CO_2_ built up gradually during the wetting phase and then declined rapidly as the soil dried, leading to clockwise hysteresis. This feature could be related to a priming of microbial respiration [18] that is lagged by root respiration in the upper soil during the dry season [59], which is beyond the scope of the present study.

By considering both soil water potential and soil moisture contents relative to soil CO_2_ concentration, we are able to see the influence of soil water on biological limitations to respiration. The saturated and near saturated soil water potential values form a near vertical line on the right hand side of the water potential graphs in Figures 4a and 4c. Variation in soil CO_2_ values for wetter soil water potential values can only be appreciated by also considering the soil moisture content graphs in Figures 4b and 4d. That is, for soil water at or near saturation, soil oxygen becomes limited, and declines in aerobic biological activities can be inferred from lower CO_2_ concentrations at these higher soil water contents. Whether this decline in aerobic biological activity is accompanied by increased anaerobic activity and increases in methane (CH_4_) concentrations requires further investigation.

After the wet-up resulting from direct precipitation on the tree island (with the wetting front moving downwards into the soil profile from the soil surface) followed by drier conditions in late March and early April (Figure 3d and e), there is evidence of wetting of the soil profile from below due to the arrival of floodwaters. The flooding period in the northern Pantanal is characterized by slow movement of large amounts of water draining from the surrounding contributing area, which slowly move down-gradient. These floodwaters arrived during late April and early May of the study period. This can be seen in the shape of the soil water content response at the 10 cm depth, which is much less abrupt than precipitation infiltration events where the soil moisture exhibits a sharp rise. The CO_2_ concentrations reached very high levels while soil CO_2_ efflux remained rather low due to water filled pore spaces slowing gas transfer from soil to the atmosphere.

During the dry season, precipitation controlled soil water dynamics and soil CO_2_ efflux. In late June and early July for example, soil respiration events accompanied changes in the soil water potential at the 10 cm depth. For one of the respiration events, no response was noted in soil water potential at 30 cm depth, with also little response in soil water content at either depth (Figure 3b, d and e). Soil respiration events triggered by precipitation that only caused changes in soil water potential at 10 cm were quite brief (e.g. July 16), whereas larger events that affected all soil water measurements lasted several days (e.g. 130 mm on July 23, which is explored greater detail in the following section). Overall, soil respiration patterns were more tightly coupled with soil water potential, particularly at the 10 cm depth. No respiration events were observed without a soil water potential response at 10 cm, though numerous CO_2_ respiration events are observed without any concomitant responses from soil water content.

### Time series analysis of soil CO_2_ efflux relative to primary controls

Wavelet coherency analysis and the conditional nonparametric spectral Granger causality analysis were used to elucidate dynamics of soil CO_2_ efflux in response to primary controls of soil water potential and soil temperature. Soil temperature was most consistently related to soil CO_2_ efflux at the daily timescale during the study (Figure 5a), although there were brief episodes of coherence at longer timescales during each of the temporal clusters. Soil water potential exhibited strong coherence at longer timescales throughout the study period (Figure 5b), suggesting that soil respiration is significantly related to soil water potential for days to weeks following precipitation events. However, the Granger causality analysis enables the causal variables to be evaluated in relation to the outcome variable independently, and while controlling for another causal variable.

Performing these analyses for each season allows an explanation of G-causality across a spectrum of timescales. During the warm-wet period, soil water potential (pF) was a significant control on soil CO_2_ efflux (F_0_) across almost the entire spectrum, and was a stronger control than soil temperature (T_s_) except for at shorter timescales (Figure 6a and 6b). The relationship between F_0_ and T_s_ found for longer timescales apparent in the wavelet coherency during the cool-dry period (Figure 5a) also showed up in the Granger causality analysis (Figure 6d). When controlling for T_s_, F_0_ was found to be more strongly G-caused by pF at the daily timescale during the cool-dry and warm-dry temporal clusters, and G-caused at a similar level for the warm wet period (0.1 for pF conditioned by T_s_, and 0.13 for T_s_ conditioned by pF, Figures 6a and 6b, respectively). pF as a control on F_0_ when conditioned by T_s_ remained significant at longer timescales in the warm-wet and cool-dry period, and was significant at the daily and shorter timescales during the warm-dry period.

## Conclusions

As the Pantanal is located within the Brazilian “agricultural frontier”, further land-use change is likely in the region, including expansion of sugarcane production for biofuels despite the relatively low agricultural potential of the region [21]. Land use practices in other areas of the Pantanal has resulted in major losses of SOC [10]. We found that the annual soil CO_2_ efflux from a tree-island environment in the Pantanal was approximately equivalent to the C stock in the upper 20 cm of soil, which suggests that SOC stocks are extremely susceptible to loss. We caution, however, that this study was unreplicated.

The frequent wetting/drying cycles result in high rates of soil carbon turnover, contributing to the non-accumulating nature of soil carbon in the Pantanal, which directly differs from the general characteristic of wetland soils with large carbon stocks and low fluxes. Soil water potential was found to be a significant control (e.g. exhibited significant G-causality) on soil CO_2_ efflux (i) across a spectrum of timescales during the warm-wet period, (ii) at daily and longer timescales during the cool-dry period, and (iii) at daily and shorter timescales during the warm-wet period.

## References

[1] Keddy PA, Fraser LH, Solomeshch AI, Junk WJ, Campbell DR, et al (2009) Wet and wonderful: the world's largest wetlands are conservation priorities. BioSci 59: 39–51.

[2] Junk W, da Cunha CN, Wantzen K, Petermann P, Strüssmann C, et al (2006) Biodiversity and its conservation in the Pantanal of Mato Grosso, Brazil. Aquat Sci 68: 278–309.

[3] van der Valk AG, Warner B (2009) The development of patterned mosaic landscapes: an overview. Plant Ecol 200: 1–7.

[4] Hanan E, Ross M (2010) Across-scale patterning of plant-soil–water interactions surrounding tree islands in Southern Everglades landscapes. Landscape Ecol 25: 463–476.

[5] Troxler Gann TG, Childers DL, Rondeau DN (2005) Ecosystem structure, nutrient dynamics, and hydrologic relationships in tree islands of the southern Everglades, Florida, USA. Forest Ecol Manag 214: 11–27.

[6] Wetzel PR, van der Valk AG, Newman S, Gawlik DE, Gann TT, et al (2005) Maintaining tree islands in the Florida Everglades: Nutrient redistribution is the key. Front Ecol Environ 3: 370–376.

[7] Batalha MA, Cianciaruso MV, Silva IA, Delitti WBC (2005) Hyperseasonal cerrado, a new brazilian vegetation form. Braz J Biol 65: 735–738. 16532198 10.1590/s1519-69842005000400021

[8] Girard P, Fantin-Cruz I, de Oliveira S, Hamilton SK (2010) Small-scale spatial variation of inundation dynamics in a floodplain of the Pantanal (Brazil). Hydrobiol 638: 223–233.

[9] Vourlitis GL, de Almeida Lobo F, Biudes MS, Rodríguez Ortíz CE, de Souza Nogueira J (2011) Spatial variations in soil chemistry and organic matter content across a invasion front in the Brazilian Pantanal. Soil Sci Soc Am J 75: 1554–1561.

[10] Maia SMF, Ogle SM, Cerri CEP, Cerri CC (2009) Soil organic carbon stock change due to land use activity along the agricultural frontier of the southwestern Amazon, Brazil, between 1970 and 2002. Global Change Biol 16: 2775–2788.

[11] Johnson MS, Lehmann J, Riha SJ, Krusche AV, Richey JE, et al (2008) CO_2_ efflux from Amazonian headwater streams represents a significant fate for deep soil respiration. Geophys Res Lett 35: L17401.

[12] Sanderman J, Chappell A (2013) Uncertainty in soil carbon accounting due to unrecognized soil erosion. Global Change Biol 19: 264–272. 10.1111/gcb.12030 23504737

[13] Raich J, Potter C (1995) Global patterns of carbon dioxide emissions from soils. Global Biogeochem Cy 9: 23–36.

[14] Lafleur PM (2009) Connecting atmosphere and wetland: trace gas exchange. Geog Compass 3: 560–585.

[15] Bond-Lamberty B, Thomson A (2010) A global database of soil respiration data. Biogeosciences 7: 1915–1926.

[16] Singh BK, Bardgett RD, Smith P, Reay DS (2010) Microorganisms and climate change: terrestrial feedbacks and mitigation options. Nat Rev Micro 8: 779–790. 10.1038/nrmicro2439 20948551

[17] Vargas R, Baldocchi DD, Allen MF, Bahn M, Black TA, et al (2010) Looking deeper into the soil: biophysical controls and seasonal lags of soil CO_2_ production and efflux. Ecol Appl 20: 1569–1582. 20945760 10.1890/09-0693.1

[18] Fierer N, Schimel JP (2003) A proposed mechanism for the pulse in carbon dioxide production commonly observed following the rapid rewetting of a dry soil. Soil Sci Soc Am J 67: 798–805.

[19] Turcu VE, Jones SB, Or D (2005) Continuous Soil Carbon Dioxide and Oxygen Measurements and Estimation of Gradient-Based Gaseous Flux. Vadose Zone J 4: 1161–1169.

[20] Cook FJ, Orchard VA (2008) Relationships between soil respiration and soil moisture. Soil Biol Biochem 40: 1013–1018.

[21] Junk WJ, Nunes da Cunha C (2005) Pantanal: a large South American wetland at a crossroads. Ecol Eng 24: 391–401.

[22] Couto E, Oliveira V (2011) The Soil Diversity of the Pantanal. In: Junk W, Da Silva C, Nunes Da Cunha C, Wantzen K, editors. The Pantanal of Mato Grosso: Ecology, biodiversity and sustainable management of a large neotropical seasonall wetland: Sofia Pensoft. 71–102.

[23] Soil Survey Staff (2010) Keys to Soil Taxonomy, 11th Ed. Washington, D.C.: USDA-Natural Resources Conservation Service.

[24] Selva EC, Couto EG, Johnson MS, Lehmann J (2007) Litterfall production and fluvial export in headwater catchments of the southern Amazon. J Tropical Ecol 23: 329.

[25] Haase R (1999) Litterfall and nutrient return in seasonally flooded and non-flooded forest of the Pantanal, Mato Grosso, Brazil. For Ecol Manage 117: 129–147.

[26] Jassal RS, Black TA, Drewitt GB, Novak MD, Gaumont-Guay D, et al (2004) A model of the production and transport of CO_2_ in soil: predicting soil CO_2_ concentrations and CO_2_ efflux from a forest floor. Ag For Meteorol 124: 219–236.

[27] Tang J, Baldocchi DD, Qi Y, Xu L (2003) Assessing soil CO_2_ efflux using continuous measurements of CO_2_ profiles in soils with small solid-state sensors. Ag For Meteorol 118: 207–220.

[28] Vaisala Oyj (2008) Vaisala CARBOCAP Carbon Dioxide Transmitter Series User's Guide. Helsinki, Finland. 44 p.

[29] Jassal R, Black A, Novak M, Morgenstern K, Nesic Z, et al (2005) Relationship between soil CO_2_ concentrations and forest-floor CO_2_ effluxes. Ag For Meteorol 130: 176–192.

[30] Webster R, Oliver MA (1990) Statistical Methods in Soil and Land Resource Survey: Oxford University Press. 328 p.

[31] Kaiser HF (1958) The varimax criterion for analytic rotation in factor-analysis. Psychometrika 23: 187–200.

[32] Dominguez E, Dawson CW, Ramirez A, Abrahart RJ (2011) The search for orthogonal hydrological modelling metrics: a case study of 20 monitoring stations in Colombia. J Hydroinform 13: 429–442.

[33] Lambrakis N, Antonakos A, Panagopoulos G (2004) The use of multicomponent statistical analysis in hydrogeological environmental research. Water Res 38: 1862–1872. 15026241 10.1016/j.watres.2004.01.009

[34] Grinsted A, Moore JC, Jevrejeva S (2004) Application of the cross wavelet transform and wavelet coherence to geophysical time series. Nonlin Processes Geophys 11: 561–566.

[35] R Development Core Team (2013) R: A language and environment for statistical computing. Version 2.15.3. Vienna, Austria: R Foundation for Statistical Computing.

[36] Gouhier TC (2012) biwavelet: Conduct univariate and bivariate wavelet analyses. R package version 0.13.

[37] Detto M, Molini A, Katul G, Stoy P, Palmroth S, et al (2012) Causality and Persistence in Ecological Systems: A Nonparametric Spectral Granger Causality Approach. Am Nat 179: 524. 22437181 10.1086/664628

[38] Hatala JA, Detto M, Baldocchi DD (2012) Gross ecosystem photosynthesis causes a diurnal pattern in methane emission from rice. Geophys Res Lett 39: L06409.

[39] Granger CWJ (1988) Some recent development in a concept of causality. J Econometrics 39: 199–211.

[40] Pasini A, Triacca U, Attanasio A (2012) Evidence of recent causal decoupling between solar radiation and global temperature. Environ Res Lett 7: 034020.

[41] Stoy P, Trowbridge A, Bauerle W (2013) Controls on seasonal patterns of maximum ecosystem carbon uptake and canopy-scale photosynthetic light response: contributions from both temperature and photoperiod. Photosynth Res doi: 10.1007/s11120-013-9799-0. 10.1007/s11120-013-9799-0 23408254

[42] Bengtsson H, Riedy J (2013) R. matlab: Read and write of MAT files together with R-to-Matlab connectivity. R package version 1.6.3.

[43] Tans P, Keeling R (2012) Trends in Atmospheric Carbon Dioxide website. Available: http://www.esrl.noaa.gov/gmd/ccgg/trends/. Accessed 2012 Sep 4.

[44] Myklebust MC, Hipps LE, Ryel RJ (2008) Comparison of eddy covariance, chamber, and gradient methods of measuring soil CO_2_ efflux in an annual semi-arid grass, Bromus tectorum. Ag For Meteorol 148: 1894–1907.

[45] Milesi de Mello J, Couto EG, Amorim R, Chig LA, Johnson MS, et al. (in review) Dina?mica do estoque de carbono no Pantanal Norte Mato-Grossense (Soil carbon stocks in the northern Pantanal). Rev Bras Cienc Solo.

[46] Cardoso EL, Naves Silva ML, de Souza Moreira FM, Curi N (2009) Atributos biológicos indicadores da qualidade do solo em pastagem cultivada e nativa no Pantanal. Pes Agropec Bras 44: 631–637.

[47] Jauhiainen J, Takahashi H, Heikkinen JEP, Martikainen PJ, Vasander H (2005) Carbon fluxes from a tropical peat swamp forest floor. Global Change Biol 11: 1788–1797.

[48] Unger S, Máguas C, Pereira JS, David TS, Werner C (2010) The influence of precipitation pulses on soil respiration – Assessing the “Birch effect” by stable carbon isotopes. Soil Biol Biochem 42: 1800–1810.

[49] Fischer T (2009) Substantial rewetting phenomena on soil respiration can be observed at low water availability. Soil Biol Biochem 41: 1577–1579.

[50] Kim DG, Vargas R, Bond-Lamberty B, Turetsky MR (2012) Effects of soil rewetting and thawing on soil gas fluxes: a review of current literature and suggestions for future research. Biogeosciences 9: 2459–2483.

[51] Maier M, Schack-Kirchner H, Hildebrand EE, Schindler D (2011) Soil CO_2_ efflux vs. soil respiration: Implications for flux models. Ag For Meteorol 151: 1723–1730.

[52] Flechard CR, Neftel A, Jocher M, Ammann C, Leifeld J, et al (2007) Temporal changes in soil pore space CO_2_ concentration and storage under permanent grassland. Ag For Meteorol 142: 66–84.

[53] Bauer J, Weihermüller L, Huisman J, Herbst M, Graf A, et al (2012) Inverse determination of heterotrophic soil respiration response to temperature and water content under field conditions. Biogeochem 108: 119–134.

[54] Orchard VA, Cook FJ (1983) Relationship between soil respiration and soil moisture. Soil Biol Biochem 15: 447–453.

[55] Lavigne MB, Foster RJ, Goodine G (2004) Seasonal and annual changes in soil respiration in relation to soil temperature, water potential and trenching. Tree Physiol 24: 415–424. 14757581 10.1093/treephys/24.4.415

[56] Parlange JY (1976) Capillary hysteresis and the relationship between drying and wetting curves. Water Res Res 12: 224–228.

[57] Pingintha N, Leclerc MY, Beasley JP Jr, Zhang G, Senthong C (2010) Assessment of the soil CO_2_ gradient method for soil CO_2_ efflux measurements: comparison of six models in the calculation of the relative gas diffusion coefficient. Tellus B 62: 47–58.

[58] Riveros-Iregui DA, Emanuel RE, Muth DJ, McGlynn BL, Epstein HE, et al (2007) Diurnal hysteresis between soil CO_2_ and soil temperature is controlled by soil water content. Geophys Res Lett 34: L17404.

[59] Baldocchi D, Tang J, Xu L (2006) How switches and lags in biophysical regulators affect spatial-temporal variation of soil respiration in an oak-grass savanna. J Geophys Res 111: G02008 doi:02010.01029/02005JG000063

